# Long Non-Coding RNA NR-133666 Promotes the Proliferation and Migration of Fibroblast-Like Synoviocytes Through Regulating the miR-133c/MAPK1 Axis

**DOI:** 10.3389/fphar.2022.887330

**Published:** 2022-04-01

**Authors:** Nanwen Zhang, Ningning Zheng, Dunxiong Luo, Duoduo Lin, Wenzhong Que, He Wang, Qiuping Huang, Juhua Yang, Jian Ye, Xiaole Chen

**Affiliations:** ^1^ The School of Pharmacy, Fujian Medical University, Fuzhou, China; ^2^ Fujian Key Laboratory of Drug Target Discovery and Structural and Functional Research, Fuzhou, China; ^3^ The Department of Physical Education, Fujian Medical University, Fuzhou, China; ^4^ Department of Rheumatology, Fuzhou No. 1 Hospital Affiliated with Fujian Medical University, Fuzhou, China; ^5^ The School of Integrative Medicine, Fujian University of Traditional Chinese Medicine, Fuzhou, China; ^6^ The Department of Orthopedics, The First Hospital of Nanping, Nanping, China

**Keywords:** long non-coding RNA (lncRNA), messenger RNA (mRNA), fibroblast-like synoviocytes (FLSs), mitogen-activated protein kinase 1 (MAPK1), rheumatoid arthritis (RA)

## Abstract

Long non-coding RNA (lncRNA) is involved in the regulation of rheumatoid arthritis (RA) and many other diseases. In this study, a new lncRNA, NR-133666, was identified to be highly expressed in the adjuvant-induced arthritis rat model using the Agilent lncRNA microarray assay. qRT-PCR verified that NR-133666 was upregulated in fibroblast-like synoviocyte of a collagen-induced arthritis (CIA) rat model. Fluorescence in situ hybridization analysis showed that NR-133666 is mainly expressed in the cytoplasm of collagen-induced arthritis FLS. MTT assay and EdU staining results showed that the proliferation of CIA FLS was inhibited after NR-133666 was knocked down, and the wound healing assay showed that the migration of CIA FLS was also suppressed. Dual luciferase detection was used to confirm the relationship among NR-133666, miR-133c and MAPK1. MAPK1 is the target gene of miR-133c, where NR-133666 acts as a sponge of miR-133c to reduce the inhibitory effect of miR-133c on MAPK1. Overexpression of NR-133666 and MAPK1 can promote the proliferation and migration of CIA FLS, and overexpression of miR-133c can reverse this phenomenon. Western blot indicated that it may be related to the ERK/MAPK signaling pathway. Collectively, we identified that lncRNA NR-133666 acted as a miR-133c sponge that can promote the proliferation and migration of CIA FLS through regulating the miR-133c/MAPK1 axis.

## Introduction

Rheumatoid arthritis (RA) is an autoimmune disease with chronic inflammation, causing pain, stiffness, and eventually functional disabilities (Culemann et al., 2019). Approximately 1% of the world population is suffering from this disease, with more than three million new cases being diagnosed each year (Zheng et al., 2015). The latest research has found that the treatment of RA is more diversified and more targeted by using monoclonal antibodies and specific drugs targeting small molecules (Davis et al., 2020). However, the etiology of RA remains unclear. The investigations of the theoretical basis for the development of novel RA therapies are urgently needed. Abnormal proliferation and migration of fibroblast-like synoviocyte (FLS) cells are important factors leading to RA (Bottini and Firestein, 2013), therefore, inhibiting its development may be an important research direction in the treatment of RA.

Recently, non-coding ribonucleic acids (ncRNAs) and small regulatory RNAs such as small interfering RNA (siRNA) and microRNA (miRNA) have been extensively studied (Song et al., 2015; Zou et al., 2018), as well as their underlying molecular mechanisms (Zou et al., 2018). Long non-coding RNA (lncRNA), playing important roles in autoimmune diseases, is a ncRNA with a length of more than 200 nucleotides (Yue et al., 2019). Previous studies have shown that overexpression of lncRNA DILC may improve RA by downregulating interleukin 6 (IL-6) and inhibiting the apoptosis of human fibroblast-like synovial cells (HFLS) (Wang et al., 2019). In addition, various lncRNAs are also involved in RA, including lncRNA H19, GAPLINC, ZFAS1, UCA1, MALAT1, C5T1, LOC100652951 and LOC100506036(Liang et al., 2019). However, the role of such lncRNAs in the pathogenesis of RA remains unclear.

MiRNA is a type of endogenous ncRNA with a length of 20–25 nucleotides, and has been shown to regulate messenger RNA (mRNA) stability and protein translation (Iorio and Croce, 2012). Studies have found that miRNA can regulate the proliferation of FLS (Cai et al., 2021). For example, miR-124a regulates the proliferation of synovial cells through the integrin beta 1 (Itgβ1)-mediated Ras-Erk1/2 signaling pathway (Wang et al., 2018). miR-133c has been reported in previous studies to be related to growth (Jia et al., 2020), development, and immune regulation (Zou et al., 2020), though the mechanism of miR-133c is still not clear in RA.

Mitogen-activated protein kinase 1 (MAPK1) can regulate the proliferation and migration of a variety of cells (Ji et al., 2019). MAPK1 has been shown to mediate the development of FLSs as an intracellular signaling molecule. Moreover, some lncRNA and mRNA can also affect the process of RA through the MAPK1 pathway (Zhu et al., 2019). For example, lncRNA NEAT1 downregulates miR-129 and miR-204 so as to regulate the process of RA through the MAPK/ERK pathway (Chen et al., 2021).

In the present study, we identified a novel lncRNA, NR-133666, which can regulate the proliferation and migration of collagen-induced arthritis (CIA) FLS. Our results clarify that NR-133666 can regulate the MAPK1 pathway by competitively inhibiting miR-133c, thereby promoting the proliferation and migration of CIA FLS.

## Materials and Methods

### Animal Preparation

Adult male Sprague-Dawley (SD) rats used in this study were 6–8 weeks old (body weight 160–180 g) (Shanghai Slack Laboratory Animal Co., Ltd.). They were cultured in the same environment that kept at room temperature 25°C and light-dark cycle for 12 h. Animals had free access to water and food. Animal experiments were carried out in accordance with the “Guidelines for the Care and Use of Laboratory Animals of the Laboratory Animal Center of Fujian Medical University” [Certificate No. SCXK (Fujian) 2016-0002], and the protocol was approved by the Animal Experiment Ethics Review Committee of Fujian Medical University (No. 2017-047).

### Animal Model Establishment

Animals were assigned to the control group and adjuvant-induced arthritis **(**AIA) groups randomly (randomization of animals were described as in a publication by Cheng T et al. (Cheng et al., 2016). Each rat in the control group was injected with 0.1 ml normal saline into the endothelium of the left posterior claw, while eight rats in the treatment group were injected with aliquots of Freund’s complete adjuvant (FCA, Chondrex. Inc. USA #200312) on designated day 0. They were euthanized (injection of pentobarbital) 27 days after AIA induction, and synovial tissue on the same side of the knee joint associated with swelling was obtained for microarray detection (Jiang et al., 2016).

### Hematoxylin and Eosin, Toluidine Blue, and Safranin O Staining

The rats were anesthetized with pentobarbital (100 mg/kg) on the 27th day. Sterile dissection was performed: the left ankle joint was isolated freshly, fixed with 4% paraformaldehyde for 24 h, and dehydrated with different concentrations of alcohol gradient, and then embedded in paraffin. The size of the tissue block was 2.0 cm × 2.0 cm ×0.3 cm. Each tissue was cut into slices (3 μm) using Leica ultra-thinslicer (Leica company, Germany), and prepared for H&E, toluidine blue, and safranin O staining (Mao et al., 2019). Under blinded conditions, the severity of inflammation and cartilage damage was scored by two independent observers. The scoring criteria were as follows: “Synovitis, 0 = normal, 1 = mild diffuse inflammatory infiltrate, 2 = moderate inflammatory infiltrate, 3 = marked inflammatory infiltrate, and 4 = severe inflammation with pannus formation. Cartilage damage and bone erosion, 0 = normal, 1 = mild loss of Safranin O staining, no obvious chondrocyte loss, 2 = moderate loss of staining with focal mild chondrocyte loss, 3 = significant staining, loss and multifocal marked chondrocyte loss, 4 = severe diffuse loss of staining, multifocal severe chondrocyte loss. Pannus, 0 = no change, 1 = pannus at two sites, 2 = pannus at four sites, 3 = pannus at more than four sites or extensive pannus at two sites” (Mukai et al., 2015).

### LncRNA and mRNA Microarray Analysis

Total RNAs were extracted from 6 cases of synoviums (3 cases from the control group and 3 cases from the model group based on H&E staining results). Quantities and qualities RNA samples were measured by NanoDrop ND-1000. RNA integrity was assessed by standard denaturing agarose gel electrophoresis. Arraystar Rat lncRNA microarrays (v2.0, containing 13,611 lncRNAs and 24,626 coding transcripts) were used to detect the expression of lncRNAs and mRNAs. The tissue preparations and microarray hybridization were performed using the Agilent Gene Expression Hybridization Kit (Agilent Technology, United States). After washing, the arrays were scanned by Agilent Microarray Scanner and finally were analyzed by Agilent Feature Extraction software (version 10.5.1.1). Differentially expressed transcripts were identified by fold-change screening at a threshold of 1.3-fold or greater, and a *p*-value < 0.05.

### Co-Expression Network

Cytoscape software (version 2.8.3, The Cytoscape Consortium, San Diego, CA, United States) was used to construct a co-expression network (CNC network) (Cui et al., 2018).

To construct the network, miRNA data targeted by the differentially expressed lncRNA NR-133666 was downloaded from the miRcode (http://www.mircode.org), and miRDB (http://mirdb.org/) and TargetScan (http://www.targetscan.org/vert_80/) were used to predict the mRNA targeted by the miRNA. Differentially expressed lncRNA and mRNA with FDR <1%, |log2 FC| ≥ 1 and Q value < 0.05 were retained to establish the competitive endogenous RNA (ceRNA) network. Finally, we used Cytoscape to construct the lncRNA-miRNA-mRNA ceRNA network (Salmena et al., 2011).

### Cell Culture

CIA FLS (BeNa Culture Collection, China) was cultured in an incubator (37°C, 5% CO_2_) in RPMI 1640 medium (Gibco, United States) supplemented with 10% fetal bovine serum (FBS, PAN, Germany). Normal rat FLS (Shanghai Yaji Biotechnology Co., Ltd.) cells were cultured in Dulbecco’s Modified Eagle Medium (DMEM, Hyclone, United States) supplemented with 10% FBS (Qishan Wang et al., 2020).

### Cell Transfection

Small interfering RNAs of lncRNA NR-133666 (si-NR-133666-1, 2, 3), small interfering RNA of MAPK1 (si-MAPK1) and their paired control (si-NC), miR-133c overexpression (miR-133c agomir) and overexpressed control (miR-133c NC) were purchased from Hanbio (Shanghai, China). The plasmids overexpressing lncRNA of lncRNA NR-133666 (pIRES NR-133666) and overexpressed control (pIRES NC) were synthesized by Zaijibio (Fuzhou, China). Cells were seeded in a 6-well plate at 1 × 10^6^ cells per well 24 h before transfection, and the transfection was started when the confluence reached 80–90%. According to the manufacturer’s instructions, we used Lipo8000™ transfection reagent (Beyotime Biotechnology #C0533) for transfection, then replaced the complete medium 6 h after transfection. All relevant sequences are listed in Supplementary Table S1.

### Quantitative Real-Time Polymerase Chain Reaction

Total RNA was extracted using TRIzol (TIANMO BIOTECH, China #TR201-50) reagent and the concentration and purity of RNA were measured using NanoDrop ND-1000 spectrophotometer. Then, the RNA underwent reverse transcription to generate complementary DNA (cDNA). Based on the directions of Hifair ^®^ III 1 st Strand cDNA Synthesis SuperMix for qPCR (YESEN, China #11141ES10), qRT-PCR was used with Hieff^®^ qPCR SYBR^®^ Green Master Mix (YESEN, China #11201ES03). 2^−ΔΔCt^ was used to assess the relative expression of genes (Shaker et al., 2019). Primers are exhibited in Supplementary Table S2.

### Dual-Luciferase Reporter Assay

Online software TargetScan 7.2 (http://www.targetscan.org/vert_72/) was used to predict the potential target genes of lncRNA NR-133666, MAPK1 and miR-133c. The 3′-untranslated region (3′-UTR) of rat lncRNA NR-133666/mutant lncRNA NR-133666 and MAPK1/mutant MAPK1 were amplified by qRT-PCR and individually subcloned into the pSI-Check2 luciferase vector (Promega, United States). We took 293T cells (ACTT, United States) and inoculated 2 × 10^4^ cells/well in a 96-well plate. After incubating overnight, we used transfection reagent (Hanbio, at a concentration of 0.8 mg/ml) to transfer wild-type (WT) or mutant (MUT) NR133666 and WT or MUT MAPK1 luciferase reporter gene plasmid and miR-133c mimic or Negative Control (NC) mimic, co-transfecting 293T cells, where each group set up was of five multiple wells. After 48 h of incubation, the luciferase activity was detected by the dual luciferase detection system (Wu et al., 2018).

### Fluorescence *in Situ* Hybridization Assay

FISH analysis using a biotin-labeled probe specific for lncRNA NR-133666 (GenePharma Co. Ltd. Shanghai, China) was performed. CIA FLS cells were fixed with 4% paraformaldehyde for 20 min, then the cells were washed twice with phosphate buffer saline (PBS). We then added protease K (20 μg/ml) to digest for 1–5 min and incubated with the FISH probe for 1 h. The nucleus was counterstained with 4,6-diamidino-2-phenylindole (DAPI). A confocal microscope (Leica Microsystems, Mannheim, Germany) was used to acquire images.

### MTT Assay

FLSs were seeded in a 96-well plate (1 × 10^4^ cells/well) and incubated overnight (37°C, 5% CO_2_). Each group was set up with six multiple wells, and we set the zero-adjustment holes, in which the zero-adjustment holes were PBS (DING GUO #BF-0011). After transfection for 12, 24, 36, 48 h, we added 20 μl of MTT solution (YESEN, China # 40201ES80) and incubated for 4 h, then aspirated the culture supernatant in the wells, added 150 μl of Dimethyl sulfoxide (DMSO) to each well. They were shook for 10 min and crystals were fully melted. The optical density at 570 nm was measured using an enzyme-linked immuno sorbent assay (ELISA) reader.

### EdU Assay

Inoculated FLSs (1 × 10^5^ cells/well) in a 24-well plate were cultured and transfected using the BeyoClick EdU-448 cell proliferation kit (Beyotime Biotechnology #C0071S) by following the manufacturer’s instructions. We incubated each well with 10 μM EdU at 37°C for 12 h. Hoechst 33,342 was used for nuclear staining, and Thermo Scientific Cellomics ArrayScan VTI HCS (Thermo Fisher Scientific, United States) was used for EdU imaging and analysis. The EdU incorporation rate was calculated based on the ratio of EdU-positive cells (green cells) to FLS-positive cells (blue cells) (Bao et al., 2020).

### Wound Healing Assay

FLSs were uniformly seeded into a 24-well plate at a density of 4 × 10^5^ cells/well. After transfection and culture, when the confluence was 90%, we used a 200 μl pipette tip to draw a straight line perpendicular to the center of the well plate. After washing with PBS twice, the cells were then cultured in a serum-free medium. We took pictures to record the scratch width at 0 h (5 pieces/hole). After 24 h in the incubator, we took pictures and recorded again at the same position. The gap distance of each monolayer was quantitatively evaluated using ImageJ. Cell migration rate was calculated as (%) = (0 h scratch area—24 h scratch area)/0 h scratch area × 100%

### Western Blot Assay

After the cells were washed twice with PBS, the cells were lysed with Radio-Immunoprecipitation Assay (RIPA) Lysis Agent (DING GUO, China) for 30 min, during which time the cells were shaken every 5 min. The protein concentration was determined using the bicinchoninic acid (BCA) protein assay kit (GLPBIO # GK10009). Protein was resolved by 12% sodium dodecyl sulphate-polyacrylamide gel electrophoresis (SDS-PAGE, DING GUO, China) and electroblotted onto a polyvinylidene difluoride membrane (PVDF). The membrane was sealed in the blocking solution (2.5 g skimmed milk powder dissolved in 50 ml TBST) for 1 h. We then probed using primary antibodies recognizing rabbit ERK (1:5,000; Affinity), p-ERK (1:5,000; CST) and β-tubulin (1:5,000; CST) overnight at 4 °C. The blot was incubated with horseradish peroxidase-conjugated secondary antibodies (1:10,000; Abcam), incubated for 2 h, and unbound secondary antibody was washed away and visualized with enhanced chemiluminescence imager (Shanghai Qinxiang Scientific Instrument Co., Ltd.).

### Statistical Analysis

The mean of all data is expressed as ± standard deviation (SD). Differences between the control group and the AIA group were analyzed using Student’s *t*-test. Multigroup comparisons are analyzed using Dunnett`s or Bonferroni`s multiple comparisons of one-way ANOVA. Spearman correlation analysis was used to detect the relationship between lncRNAs and mRNA. Two-tailed *p* < 0.05 was used to demonstrate that the difference was statistically significant.

## Results

### Identification of Differentially Expressed lncRNAs and mRNAs in AIA Animals

**FIGURE 1:**
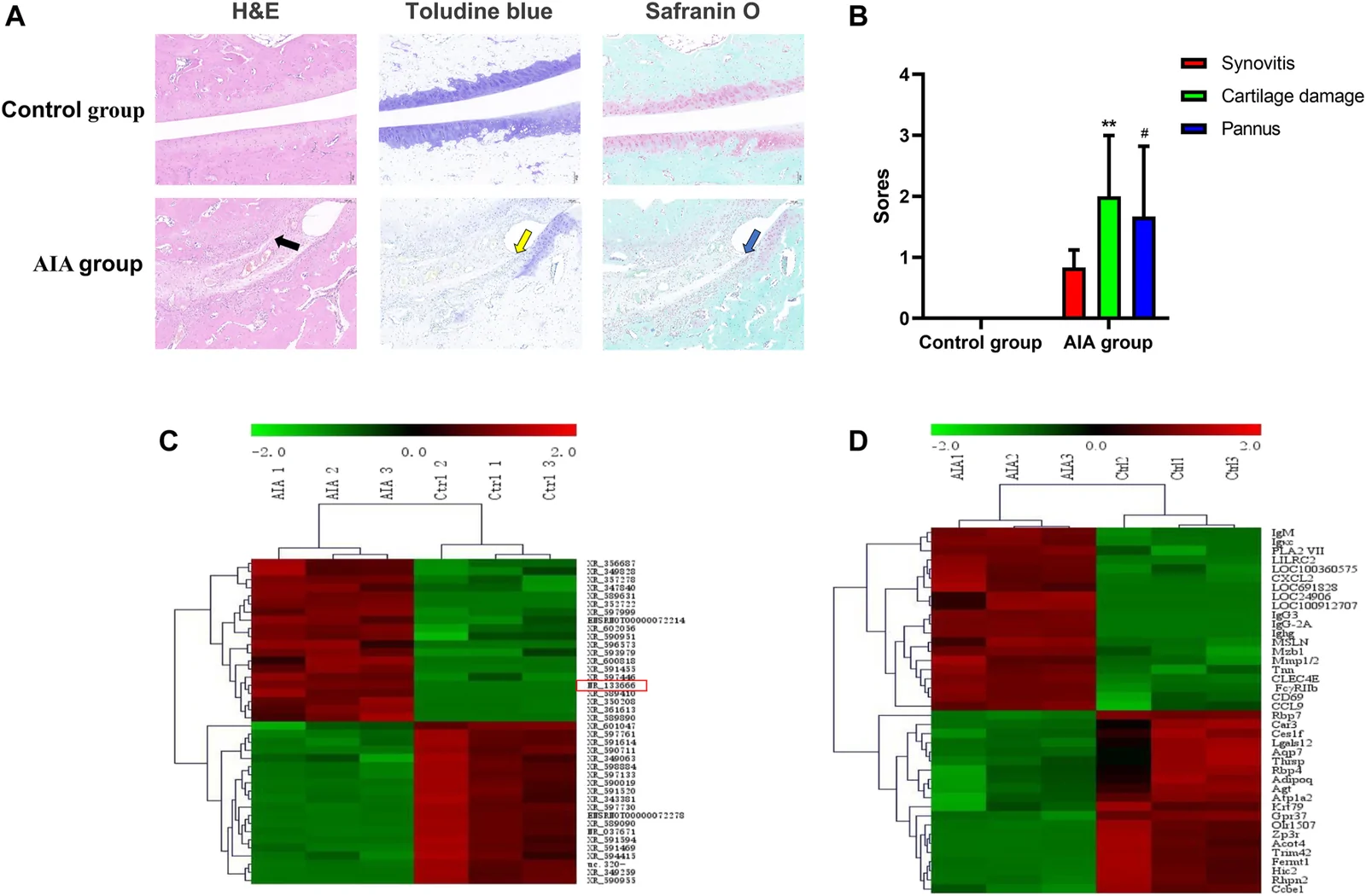
Pathological and morphological changes and the different expression analysis of lncRNA and mRNA in synovial tissue from the control and AIA model group. **(A)** Compared with the control group, H&E staining of knee joint sections showed severe articular cartilage damage, adhesion and lymphocyte infiltration in the AIA group (black arrow); toluidine blue and safranin O staining showed that a large area of staining was lost and a large amount of chondrocytes was lost (yellow arrow), and bone damage was serious (blue arrow). **(B)** The quantitative analysis showed severe synovitis, cartilage damage and pannus of AIA model group (*n* = 3). Compared with controls, **p < 0.01, ^#^ *p* < 0.05. **(C)** Heat maps show the top 20 significantly upregulated and downregulated lncRNAs. lncRNA NR-133666 was enhanced in the AIA group. **(D)** Heat maps show the top 20 significant upregulated and downregulated mRNAs.

H&E staining of the AIA specimen displayed that the inflammatory cell infiltration in the articular cartilage surface as well as the cartilage erosion (swelling, exfoliation, pannus tissue formation) and pannus formation (Figure 1A). Statistical integration showed that the scores of AIA group were more than the control group, which indicated that the AIA model was successfully established (Figure 1B). A total of 13,611 lncRNAs were detected, and the Agilent lncRNA microarray identified 2009 differentially expressed lncRNAs with multiple variation cued off to 2.0 (1,282 upregulated and 727 decreased, *p* < 0.05). The 20 mRNAs with the most significant upregulation and downregulation are shown (Figure 1C). Compared with the control group, we identified 4,547 differentially expressed mRNAs in AIA animals using mRNA microarray, with a multiple cutoff of 2.0 (2,828 upregulated and 1719 decreased, *p* < 0.05). The 20 mRNAs with the most significant upregulation and downregulation are shown (Figure 1D).

### LncRNA-mRNA CNC

**FIGURE 2:**
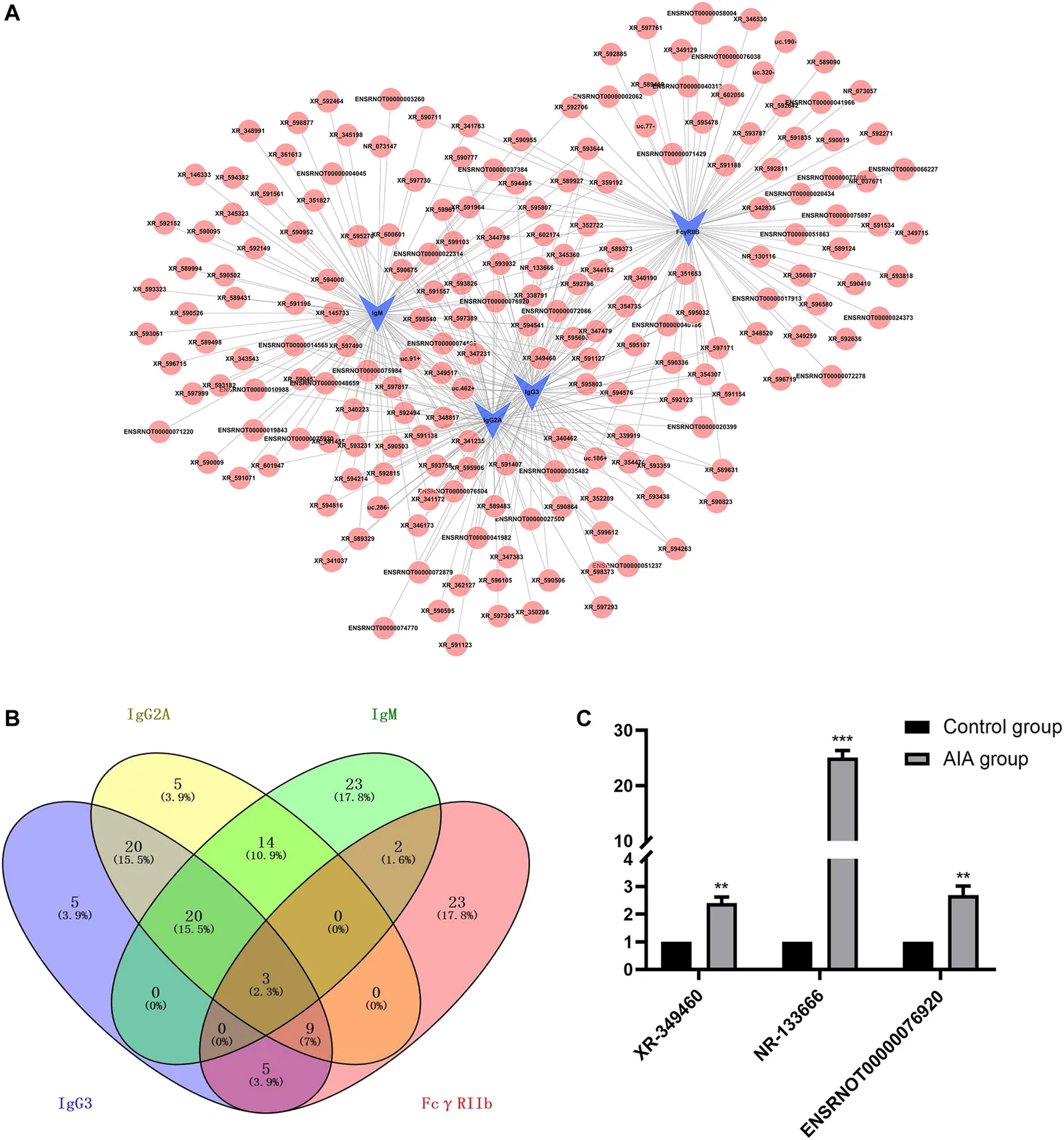
lncRNA-mRNA co-expression networks and real-time quantitative PCR validation. **(A)** Four verified co-expression networks of mRNAs and their associated lncRNAs. The blue dots represent mRNAs and red dots represent their correlated lncRNAs. There were 309 positive interactions (linked with continuous lines) and 66 negative interactions (linked with dotted lines). The absolute value of correlation coefficients of all the directly linked pairs exceeded 0.990. **(B)** Venn diagram illustrating the intersections of lncRNAs with Pearson correlation coefficients equal to or higher than 0.992, using 4-way Venn Diagram Generator. The common intersection result is upregulated lncRNA XR-349460, NR-133666 and ENSRNOT00000076920. **(C)** The qRT-PCR validation of the three selected lncRNA. Results were similar to that of lncRNA microarray.

We first constructed a network of lncRNA and mRNA co-expression based on four verified differentially expressed mRNAs [Immunoglobulin G (IgG)3, IgG2A, IgM and FcγRIIb] and 375 interacted lncRNAs. The results showed that IgG3 was correlated with 88 lncRNAs (84 positive correlation, 4 negative correlation); IgM was correlated with 97 lncRNAs (77 positive correlation, 20 negative correlation); IgG2A was correlated with 101 lncRNAs (97 positive correlation, 4 negative correlation); and FcγRIIb was correlated with 89 lncRNAs (51 positive correlation, 38 negative correlation) (Figure 2A). We selected lncRNAs with a Pearson correlation coefficient ≥0.992, and then used the 4-way Venn Diagram Generator (https://bioinfogp.cnb.csic.es/tools/venny/index.html) to construct the network in each of the above groups to select lncRNAs related to the pathogenesis of RA molecules. The common intersection result suggested upregulation of lncRNA XR-349460 (LOC102556662, chromosome14, fold change 5.61), lncRNA NR-133666 (LOC102554317, chromosome11, fold change 38.10) and lncRNA ENSRNOT00000076920 (LOC100911498, chromosome X, fold change 3.82) (Figure 2B). XR-349460, NR-133666 and ENSRNOT00000076920 were overexpressed in AIA synovial tissues, as verified further by qRT-PCR. Among them, the expression difference of NR-133666 was more than 38-fold greater (Figure 2C). Hence, NR-133666 was selected for the subsequent functional and molecular mechanism experiments.

### LncRNA NR-133666 is Highly Expressed in the Cytoplasm of CIA FLS and Promotes CIA FLS Proliferation and Migration

**FIGURE 3:**
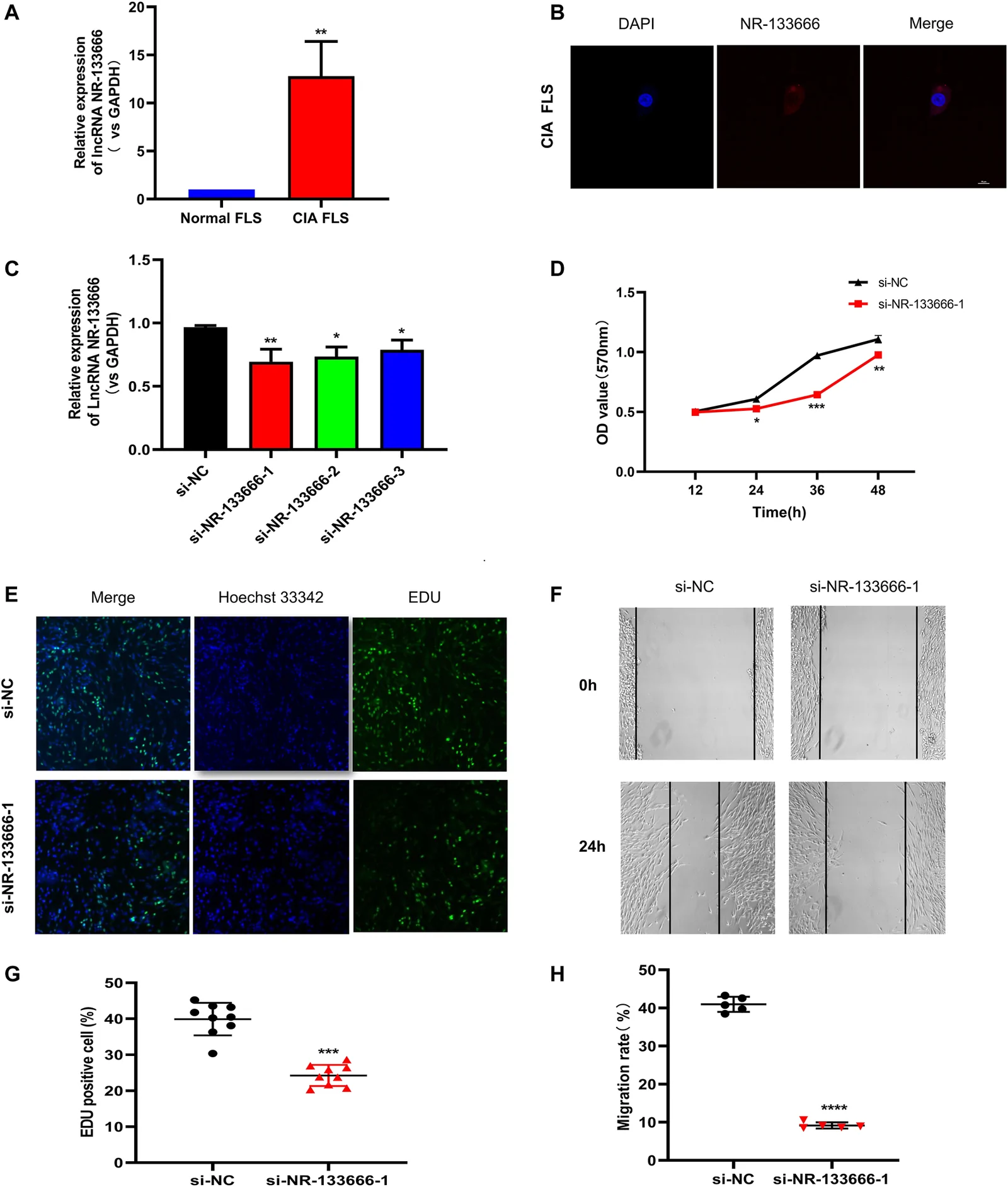
lncRNA NR-133666 is highly expressed in the cytoplasm of CIA FLS and promotes CIA FLS proliferation and migration. **(A)** qRT-PCR showed that NR-133666 was increased in CIA FLS. **(B)** RNA-FISH localization of NR-133666 in CIA FLS. **(C)** qRT-PCR showed that NR-133666 was reduced in si-NR-133666-transfected macrophages, among them si-NR-133666-1 was the most obvious. **(D)** MTT showed that the proliferation of si-NR-133666-1 transfected CIA FLS was reduced. **(E,F)** EdU assay showing CIA FLS proliferation decreased after transfection with si-NR-133666-1 (100×). **(G,H)** The migratory ability of CIA FLS cells was weakened after transfection with si-NR-133666-1, measured by wound healing assay (100×). Compared with controls, *p < 0.05, **p < 0.01, ***p < 0.001, ****p < 0.0001.

The expression level of lncRNA NR-133666 in CIA FLS was increased compared with normal FLS (Figure 3A). Moreover, a FISH assay revealed that lncRNA NR-133666 was localized mostly in the cytoplasm of CIA FLS (Figure 3B).

In order to evaluate the function of NR-133666 in CIA FLS, three siRNAs targeting the junction of NR-133666 were designed. qRT-PCR indicated that the expression levels of NR-133666 decreased significantly after transfection with siRNAs, where si-NR-133666-1 exhibited the best effect, so that was selected in subsequent experiments (Figure 3C). The result of MTT tests, which were used to detect the level of cell proliferation, pointed out that knockdown of NR-133666 reduced the proliferation of CIA FLS compared with the si-NC group (Figure 3D). The EdU assay was employed to determine the role of NR-133666 in cell proliferation. The number of proliferating cells (green fluorescence) was reduced significantly after transfection with si-NR-133666-1 (Figures 3E,F). Wound healing assay was applied to detect the migration levels. After knocking down NR-133666, the migration of CIA FLS was significantly inhibited compared with the si-NC group (Figures 3G,H).

### MiR-133c had a Low Expression in CIA FLS and lncRNA NR-133666 May be a Sponge of miR-133c

**FIGURE 4:**
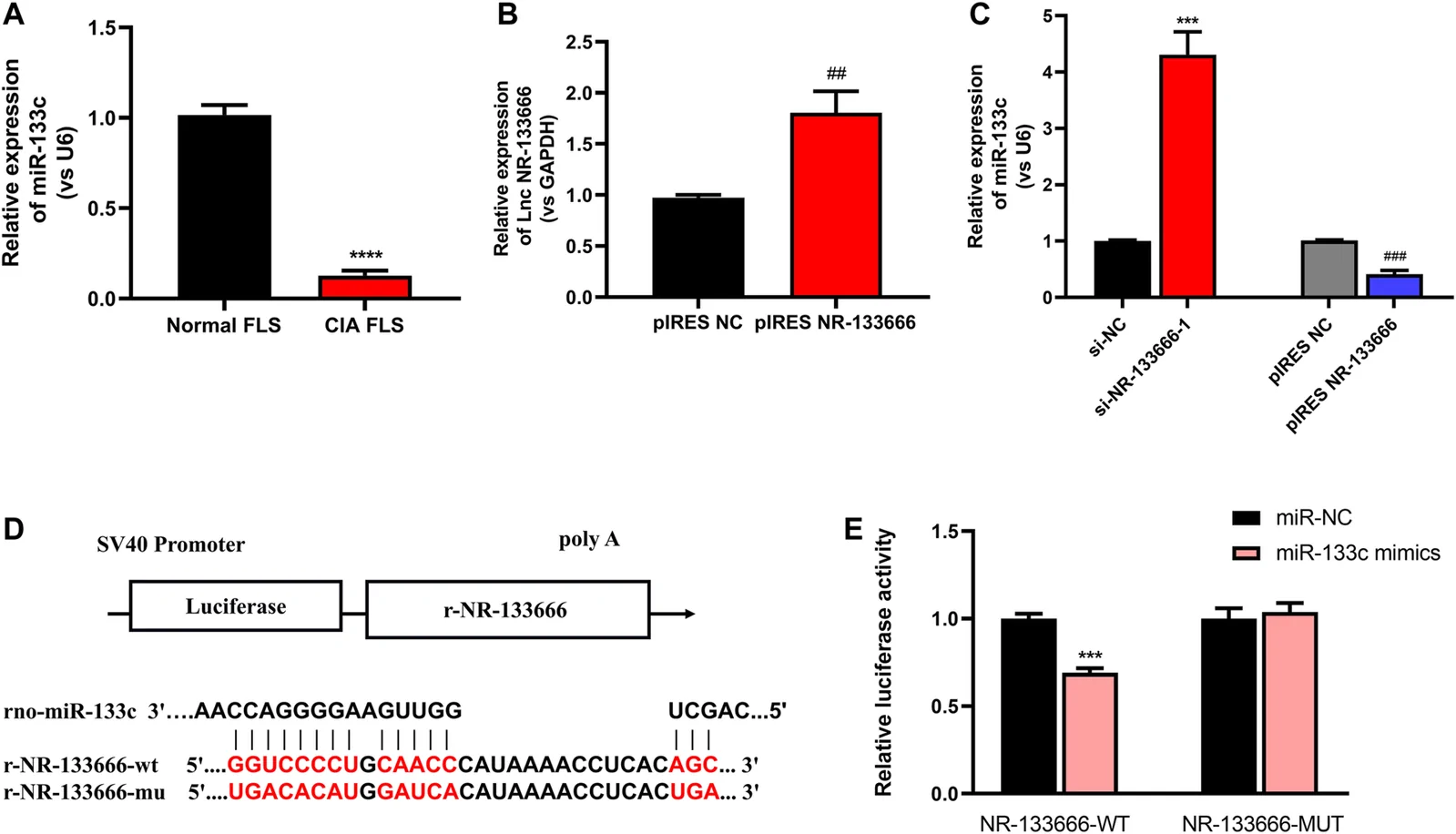
lncRNA NR-133666 behaves like a sponge for miR-133c. **(A)** qRT-PCR showed that miR-133c was increased in CIA FLS. **(B)** qRT-PCR showed that NR-133666 was increased in pIRES NR-133666-transfected CIA FLS. **(C)** The expression of miR-133c in si-NR-133666–1 and pIRES NR-133666 groups. **(D)** The predicted binding site between miR-133c and NR-133666. **(E)** Co-transfection of lncRNA NR-133666 WT and miR-133c mimics decreased luciferase activity. Compared with controls, ***p < 0.001, ****p < 0.0001; compared with pIRES NC, ^##^ *p* < 0.01, ^###^ *p* < 0.001.

qRT-PCR showed that low expression of miR-133c was detected in CIA FLS (Figure 4A). The transfection efficiency of overexpression of NR-133666 was measured, and the results showed that the expression of NR-133666 was upregulated after transfection (Figure 4B). qRT-PCR results showed that miR-133c was highly expressed after the transfection of si-NR-133666-1, while expression of miR-133c was low after the overexpression of NR-133666 (pIRES NR-133666) (Figure 4C). Bioinformatics demonstrated that mir-133c was the target of NR-133666 (Figure 4D). Dual luciferase reporter assay showed that miR-133c mimics decreased the luciferase activity of the WT NR-133666 vector, but not that of the MUT NR-133666 vector (Figure 4E). Therefore, we confirmed that lncRNA NR-133666 was a sponge of miR-133c, and speculated that NR-133666 may affect the function of CIA FLS by regulating miR-133c.

### LncRNA NR-133666 Regulates the Proliferation and Migration of CIA FLS by Regulating miR-133c

**FIGURE 5:**
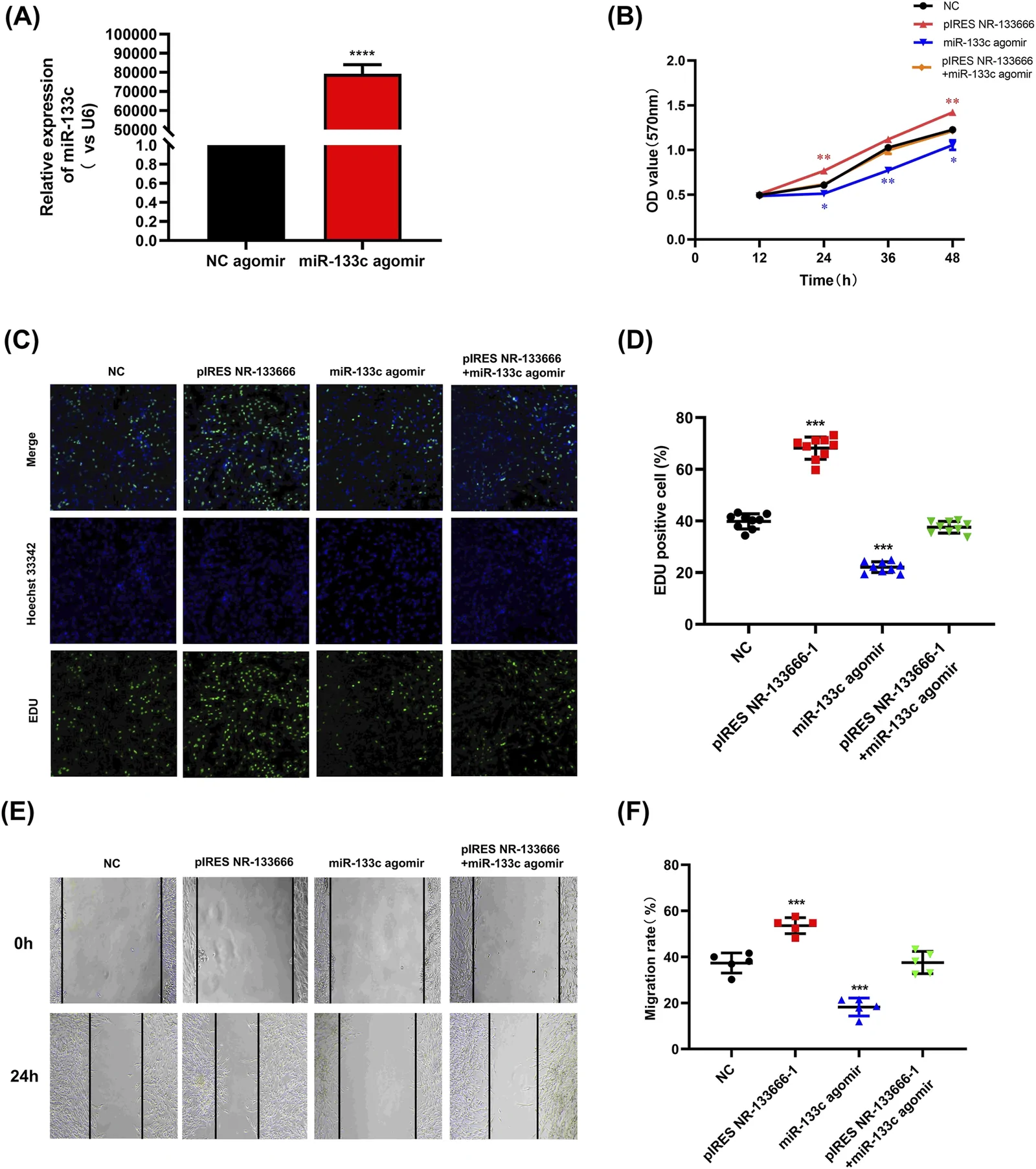
. lncRNA NR-133666 regulates the proliferation and migration of CIA FLS by regulating miR-133c. **(A)** qRT-PCR showed that miR-133c was increased in miR-133c agomir-transfected CIA FLS. **(B)** MTT was performed to verify the effect of miR-133c overexpression, NR-133666 overexpression and co-expression groups on CIA FLS proliferation. **(C,D)** EdU assay showed CIA FLS proliferation after transfecting pIRES NR-133666, miR-133c and pIRES NR- 133666 +miR-133c groups (100×). **(E,F)** Wound healing assay showed the migratory ability of CIA FLS cells (100×). Compared with controls, *p < 0.05, **p < 0.01, ***p < 0.001, ****p < 0.0001.

qRT-PCR results showed that the expression of miR-133c was greatly increased after using the miR-133c agomir (Figure 5A). MTT assay showed that cell proliferation level was increased by pIRES NR-133666 and the condition was offset with the addition of miR-133c mimics (Figure 5B). EdU experiment further verified that the number of proliferating cells increased significantly after transfection with pIRES NR-133666 and the cell quantity was restored by the miR-133c agomir (Figures 5C,D). Wound healing assay showed that the upregulation of NR-133666 significantly promoted the migration abilities of CIA FLS and overexpression of miR-133c could inhibit the migration of CIA FLS. Moreover, miR-133c agomir could counteract the effect of pIRES NR-133666 (Figures 5E,F).

### MAPK1 Was the Target Gene of miR-133c

**FIGURE 6:**
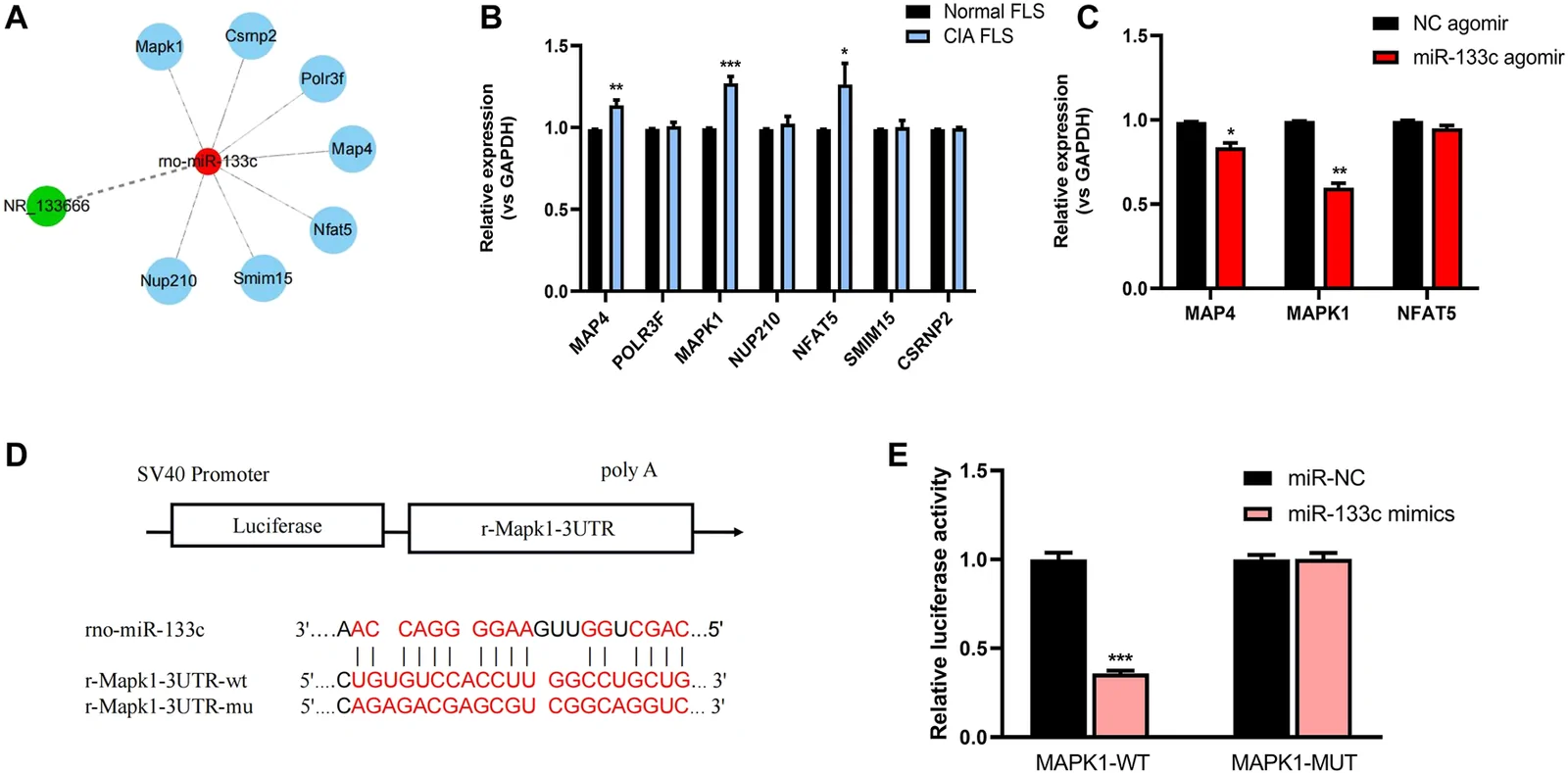
MAPK1 was the target gene of miR-133c. **(A)** Targeted mRNAs of lncRNA NR-133666–miR-133c axis scanned in databases of miRDB and TargetScan. **(B)** qRT-PCR showed the expression of mRNA in CIA FLS, compared with normal FLS. **(C)** qRT-PCR showed the expression of MAP4, MAPK1 and NFAT5 mRNAs in miR-133c agomir-transfected CIA FLS, compared with NC agomir. **(D)** Complementary bases of miR-133c and MAPK1. **(E)** Co-transfection of MAPK1 WT and miR-133c mimics decreased luciferase activity. Compared with controls, *p < 0.05, **p < 0.01, ***p < 0.001.

The potential lncRNA NR-133666–miR-133c–mRNA network was scanned in databases of miRDB and TargetScan (Figure 6A). qRT-PCR results showed that MAP4, MAPK1 and Nuclear Factor of Activated T-cells 5 (NFAT5) are overexpressed in CIA FLS relative to normal FLS (Figure 6B). Further analysis showed that after applying the miR-133c mimic, only the expression of MAP4 and MAPK1 was downregulated, which demonstrated that MAP4 and MAPK1 may be the target genes of miR-133c (Figure 6C). Previous studies found that MAPK1 was an important target for the treatment of RA (Wenxiang Wang et al., 2020). Bioinformatics and dual luciferase reporter assay presented that MAPK1 was the target of miR-133c (Figure 6D). The results demonstrated that in the presence of miR-133c mimics, the luciferase activity from the WT construct decreased. However, the mimics had no effect on the luciferase activity from the MUT construct (Figure 6E). The results indicate that MAPK1 is the target gene of miR-133c.

### lncRNA NR-133666 Promoted CIA FLS Proliferation and Migration Through miR-133c/MAPK1 Axis

**FIGURE 7:**
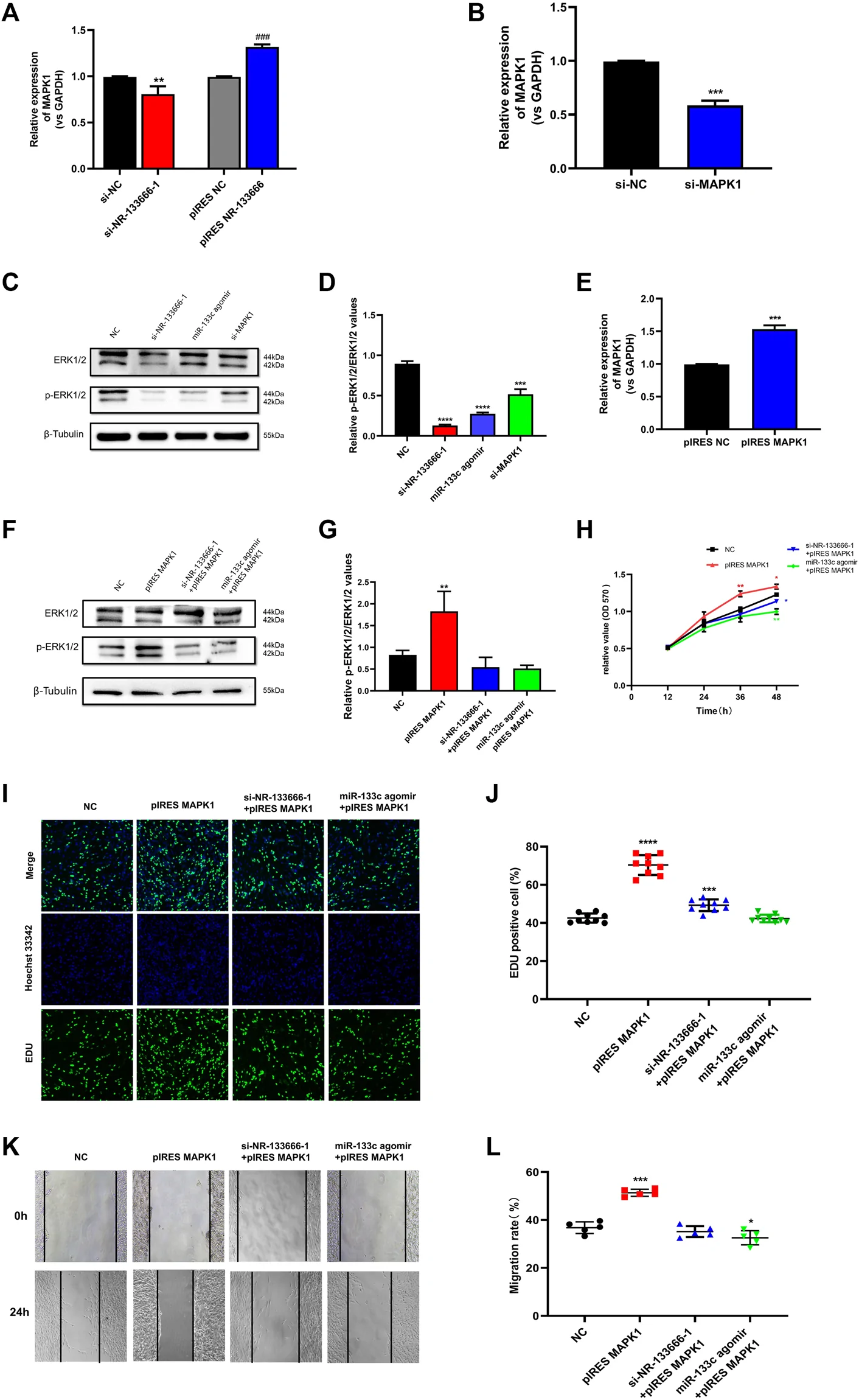
lncRNA NR-133666 promoted CIA FLS proliferation and migration through miR-133c/MAPK1 axis. **(A)** MAPK1 expression in si-NR-133666–1 and pIRES NR-133666 groups. **(B)** qRT-PCR detected the transfection effect of si-MAPK1. **(C,D)** The Western blot analysis showed that knockdown NR-133666 and MAPK1 or overexpression miR-133c can reduce the expression of p-ERK1/2 in CIA FLS. **(E)** qRT-PCR showed the transfection effect of pIRES MAPK1. **(F,G)** The protein expression of p-ERK1/2 was detected by Western blot after transfecting pIRES MPAK1, si-NR-133666-1+pIRES MAPK1 and miR-133c agomir + pIRES MAPK1 in CIA FLS. **(H–L)** Functional rescue experiments were performed on proliferation and validated migration in pIRES MPAK1, si-NR-133666-1+pIRES MAPK1 and miR-133c agomir + pIRES MAPK1 (100×). Compared with controls, *p < 0.05, **p < 0.01, ***p < 0.001, ****p < 0.0001; compared with pIRES NC, ^###^ *p* < 0.001.

qRT-PCR results further indicated that after knocking down or overexpressing NR-133666, the expression of MAPK1 was also positively regulated (Figure 7A). The ERK/MAPK pathway may be affected by lncRNA NR-133666. Transfection efficiency verification was used for further experiments. The expression of MAPK1 was downregulated after transfection of si-MAPK1 (Figure 7B). Then, we detected the phosphor-ERK1/2 (p-ERK1/2) and total ERK protein levels by Western blot in CIA FLS that knocked down NR-133666 and MAPK1 or overexpressed miR-133c, respectively. The results showed the levels of p-ERK1/2 was dramatically decreased (Figures 7C,D). qRT-PCR verified the transfection efficiency of pIRES MAPK1 for further experiments (Figure 7E). Western blot analyses suggested that the phosphorylation level of ERK1/2 was increased following MAPK1 overexpression, and overexpression of MAPK1 could partially reverse the phosphorylation of ERK1/2 by si-NR-133666–1 and miR-133c agomir (Figures 7F,G).

MTT assay showed that CIA FLS proliferation level was increased in the pIRES MAPK1 group. The condition was offset with the addition of si-NR-133666-1 and miR-133c (Figure 7H). The EdU experiment also further verified the above finding (Figures 7I,J). Wound healing test showed that the upregulation of MAPK1 significantly promoted the migration ability of CIA FLS, though knocked down NR-133666 and added miR-133c agomir could inhibit this phenomenon (Figures 7K,L).

The above results showed that lncRNA NR-133666 may promote CIA FLS proliferation and migration through the miR-133c/MAPK1 axis. At the same time, these genes may also regulate the proliferation and migration of CIA FLS by activating the MAPK/ERK pathway.

## Discussion

**FIGURE 8:**
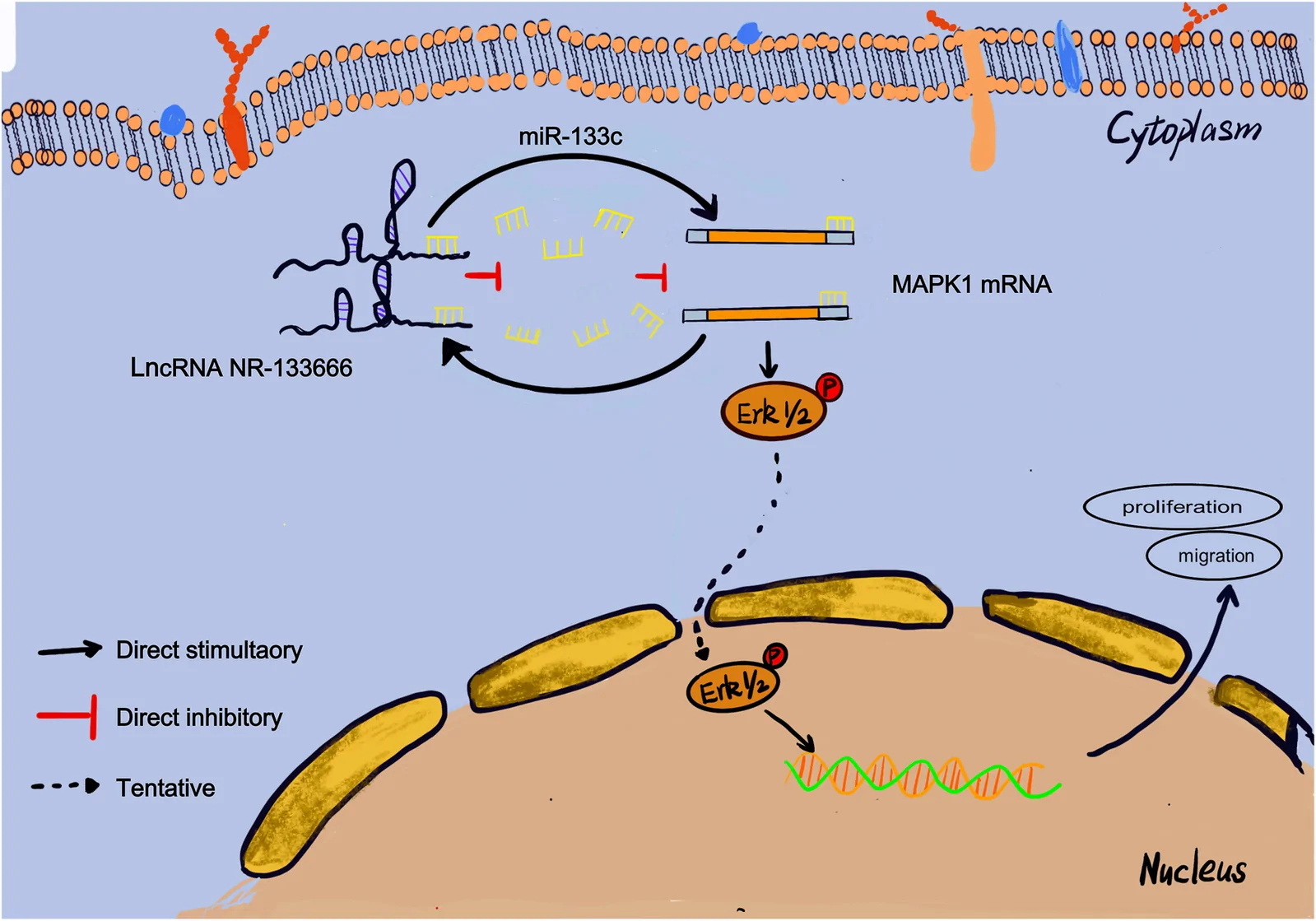
Mechanistic model. This schematic diagram represents the potential molecular mechanism that lncRNA NR-133666 promotes the proliferation and migration of CIA FLS and indirectly upregulates MAPK1 and ERK phosphorylation to activate ERK signaling by regulating miR-133c.

RA is a common autoimmune disease with joint damage as the symptom (Wu et al., 2015). In recent decades, people have conducted extensive studies on the expression profiles of genes related to the pathogenesis of RA (Rahimizadeh et al., 2021). Previous studies have shown the importance of lncRNA in the pathogenesis of RA (Sigdel et al., 2015). However, there are only a few studies on the role of lncRNAs in regulating the immune responses in RA. Here, we used the Arraystar Rat lncRNA/mRNA microarray to analyze AIA rats and found that the level of immunoglobulin correlates with the expression of lncRNA. According to the microarray expression profiles, we found 4,547 differentially expressed mRNAs, including 2,828 upregulated and 1719 downregulated mRNAs. We identified a new lncRNA, NR-133666, which is upregulated in CIA FLS and AIA rats and can promote the proliferation and migration of CIA FLS. In terms of mechanism, NR-133666 can act as a sponge of miR-133c, where MAPK1 is the target of miR-133c. When the level of miR-133c was reduced, its inhibitory effect on MAPK1 was reduced by activating the MAPK/ERK pathway so as to ultimately promote proliferation and migration. This indicated that the lncRNA NR-133666/miR-133c/MAPK1 axis plays an important role in regulating the proliferation and migration of CIA FLS (Figure 8).

Various pro-inflammatory cytokines can promote the proliferation, migration and adhesion of joint synovial cells, which have been shown to be the key to the clinical progression of RA (Malemud, 2018). Therefore, it is imperative to accurately identify the relevant molecular mechanisms that promote the proliferation and migration of FLSs(Croia et al., 2019). The lncRNA NR-133666 and miR-133c found in this study can provide new insight into the precise inhibition of FLSs in RA.

More and more studies have reported that abnormally expressed lncRNAs can be used as ceRNA to regulate miRNAs and play a key role in the occurrence and development of autoimmune diseases (Chen et al., 2019). In this study, we showed the direct correlation between lncRNA NR-133666, MAPK1 and miR-133c through biological information analysis, dual luciferase experiment and co-transfection. Among them, lncRNA NR-133666 may be used as ceRNAs to regulate miRNA-133c, thereby regulating the progression of CIA FLS. Both NR-133666 and miR-133c are new and there is no report indicating their role in RA. Overexpression of NR-133666 and inhibition of miR-133 can promote the proliferation and migration of CIA FLS. Therefore, they may become a new target for the treatment of RA.

MAPK signaling pathway usually regulates inflammatory response, proliferation and migration of cells, and plays a prominent role in RA. According to research reports, activating the MAPK/ERK pathway can promote the proliferation and migration of RA FLS (Liu et al., 2018). MAPK1 is an important component of the MAPK/ERK pathway (Hong et al., 2018). In our study, overexpression of MAPK1 also promoted the proliferation and migration of CIA FLS, which was regulated by NR-133666 and miR-133c. It showed that lncRNA NR-133666/miR-133c mediates the progression of CIA FLS through the MAPK1 signaling pathway.

In conclusion, this study discovered a differentially expressed lncRNA, NR-133666, and demonstrated that it can affect the proliferation and migration of CIA FLS cells through the lncRNA NR-133666/miR-133c/MAPK1 axis. Our findings indicated that the lncRNA NR-133666 and miR-133c can be potential therapeutic targets for the prevention and treatment of human RA.

## Data Availability

The original contributions presented in the study are included in the article/Supplementary Material, further inquiries can be directed to the corresponding authors.
