# Correction: Long non-coding RNA NR-133666 promotes the proliferation and migration of fibroblast-like synoviocytes through regulating the miR-133c/MAPK1 axis

**DOI:** 10.3389/fphar.2026.1950778

**Published:** 2026-09-23

**Authors:** Nanwen Zhang, Ningning Zheng, Dunxiong Luo, Duoduo Lin, Wenzhong Que, He Wang, Qiuping Huang, Juhua Yang, Jian Ye, Xiaole Chen

**Affiliations:** 1 The School of Pharmacy, Fujian Medical University, Fuzhou, China; 2 Fujian Key Laboratory of Drug Target Discovery and Structural and Functional Research, Fuzhou, China; 3 The Department of Physical Education, Fujian Medical University, Fuzhou, China; 4 Department of Rheumatology, Fuzhou No. 1 Hospital Affiliated with Fujian Medical University, Fuzhou, China; 5 The School of Integrative Medicine, Fujian University of Traditional Chinese Medicine, Fuzhou, China; 6 The Department of Orthopedics, The First Hospital of Nanping, Nanping, China

**Keywords:** long non-coding RNA (lncRNA), messenger RNA (mRNA), fibroblast-like synoviocytes (FLSs), mitogen-activated protein kinase 1 (MAPK1), rheumatoid arthritis (RA)

There was a mistake in Figure 5E as published. For the wound healing assay, multiple fields of cell culture wells were captured simultaneously. The overlapping issue between the 24 h image of the pIRES NR-133666 + miR-133c agomir group and the NC group in Figure 5E was an unintentional error caused by incorrect image file naming after photography and improper selection of images during figure compilation. Alternative representative 0 h and 24 h fields from the pIRES NR-133666 + miR-133c agomir group have been selected for correction. The corrected Figure 5 appears below.

**FIGURE 5:**
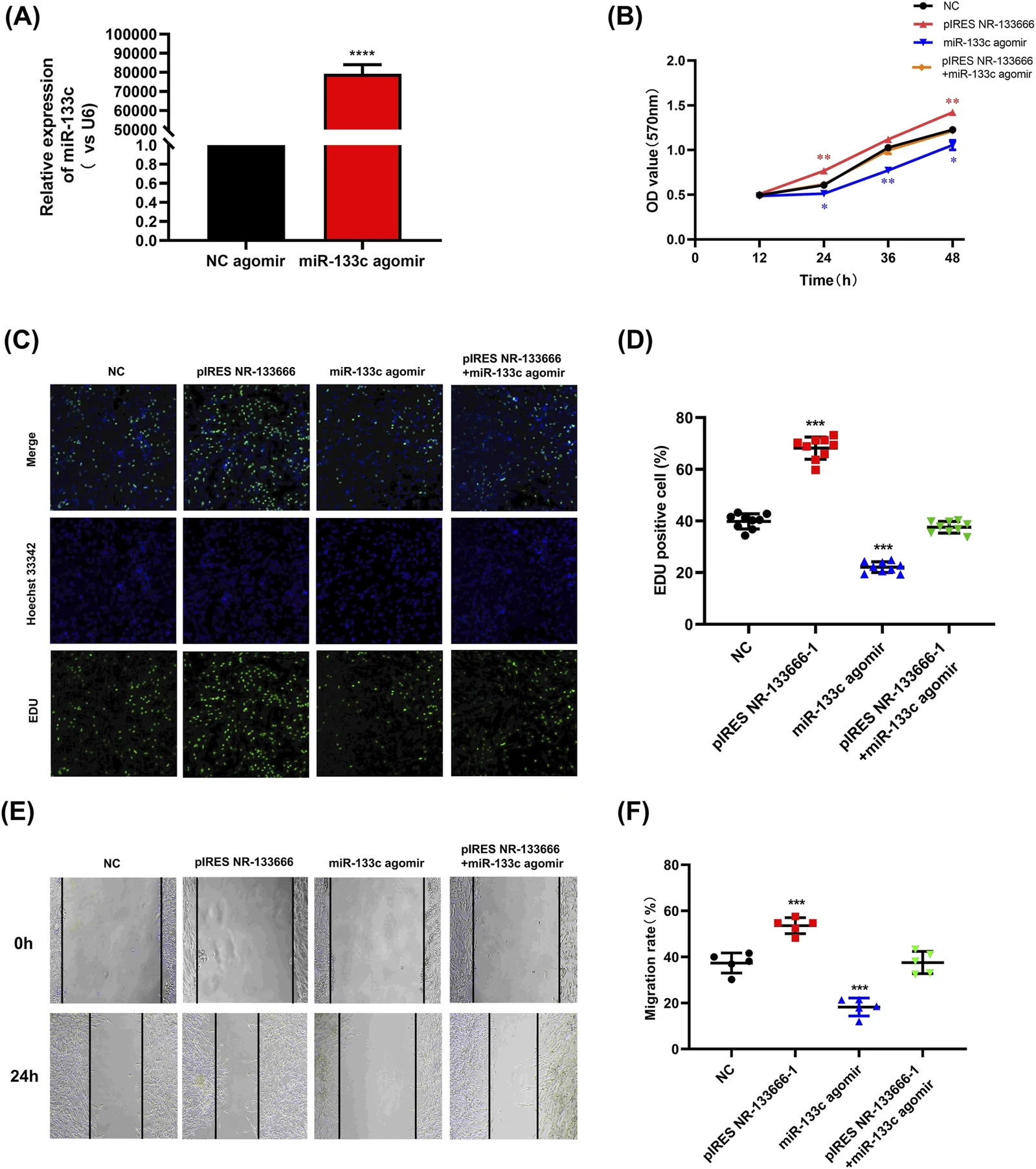
lncRNA NR-133666 regulates the proliferation and migration of CIA FLS by regulating miR-133c. **(A)** qRT-PCR showed that miR-133c was increased in miR-133c agomir-transfected CIA FLS. **(B)** MTT was performed to verify the effect of miR-133c overexpression, NR-133666 overexpression and co-expression groups on CIA FLS proliferation. **(C,D)** EdU assay showed CIA FLS proliferation after transfecting pIRES NR-133666, miR-133c and pIRES NR-133666 +miR-133c groups (100×). **(E,F)** Wound healing assay showed the migratory ability of CIA FLS cells (100×). Compared with controls, *p < 0.05, **p < 0.01, ***p < 0.001, ****p < 0.0001.

The original version of this article has been updated.

