# Supplementary material for: Long Non-Coding RNA NR-133666 Promotes the Proliferation and Migration of Fibroblast-Like Synoviocytes Through Regulating the miR-133c/MAPK1 Axis
Source: Front Pharmacol. 2022 Apr 1;13:887330. doi: 10.3389/fphar.2022.887330 (PMC9012539; doi:10.3389/fphar.2022.887330)
Supplement: Supplementary file 1 [file Table1.DOCX]

**Supplementary Table S1.** Sequences used in this study.

| **Gene Name** | **Primer sequence** |
| --- | --- |
| si-NC  si-NR-133666-1  si-NR-133666-2  si-NR-133666-3  si-MAPK1 | Forward: UUCUCCGAACGUGUCACGU TT  Reverse: ACGUGACACGUUCGGAGAA TT  Forward: GCCAGUAUCUUGAUCAUAU TT  Reverse: AUAUGAUCAAGAUACUGGC TT  Forward: GGCCAGACCAACAGUAAAU TT  Reverse: AUUUACUGUUGGUCUGGCC TT  Forward: GGUGAGGCUUCUGAGUAAA TT  Reverse: UUUACUCAGAAGCCUCACC TT  Forward: CCCUCACAAGAGGAUUGAA TT  Reverse: UUCAAUCCUCUUGUGAGGG TT |
| miR-133c agomir | Forward: CAGCUGGUUGAAGGGGACCAA  Reverse: UUGGUCCCCUUCAACCAGCUG |
