# Supplementary material for: Long Non-Coding RNA NR-133666 Promotes the Proliferation and Migration of Fibroblast-Like Synoviocytes Through Regulating the miR-133c/MAPK1 Axis
Source: Front Pharmacol. 2022 Apr 1;13:887330. doi: 10.3389/fphar.2022.887330 (PMC9012539; doi:10.3389/fphar.2022.887330)
Supplement: Supplementary file 2 [file Table2.DOCX]

**Supplementary Table S2.** Primer sequences for qRT-PCR.

| **Gene Name** | **Primer sequence** |
| --- | --- |
| LncRNA XR 349460 | Forward: GCACGTGTAAATTTTCCTGATGGG |
|  | Reverse: AGAGGAGTGGAAGTAGGTCTGT |
| LncRNA NR 133666 | Forward: AACCTCACAGCGGCACTACA |
|  | Reverse: GGGTCTGGCTTTAAGATGGC |
| LncRNA ENSRNOT00000076920 | Forward: ATAGCCAGAGCAATGACGGG |
| miR-133c  MAPK1  Map4  POLR3F  NUPU210  NFAT5  CSRNP2  SMIM15 | Reverse: TACCATGTGGGGAGTCTGGT  Forward: CCGCAGCTGGTTGAAGGG  Reverse: AGTGCAGGGTCCGAGGTATT  Forward: AATAAGGTGCCGTGGAACAG  Reverse: GCTCATCTTGGGTCATAATACT  Forward: AGAACTGAAGGTGCTGGATGA  Reverse: TTGGGCTGATGTGGAGTAGG  Forward: GGCATCAGTAAGGTAGAGTTGTC  Reverse: TTCCTTGGCAGCAATGATAGTC  Forward: TTGACTTCCCTGCTCGTGAC  Reverse: GATGTACGGCTGCTAGGCAT  Forward: GTCCTACGCCATCTGAGTCA  Reverse: GAGTGTTACTTGTTGCTCTTGAGA  Forward: TTACCTCACTGGTGGCTTGC  Reverse: CGTTCAGGCTCCTTACGGTT  Forward: GAGTATGTTGTGGAGTGGGC  Reverse: TCTGGCTTCAATCATCTTGGC |
| GAPDH | Forward: GCTCTCTGCTCCTCCCTGTTCT |
|  | Reverse: TGGTAACCAGGCGTCCGATA |
| U6 | Forward: CTCGCTTCGGCAGCACATATACT  Reverse: AGTGCAGGGTCCGAGGTATT |
